# Evaluating Virtual Planning Accuracy in Bimaxillary Advancement Surgery: A Retrospective Study Introducing the Planning Accuracy Coefficient

**DOI:** 10.3390/jcm14103527

**Published:** 2025-05-18

**Authors:** Paweł Piotr Grab, Michał Szałwiński, Maciej Jagielak, Jacek Rożko, Dariusz Jurkiewicz, Aldona Chloupek, Maria Sobol, Piotr Rot

**Affiliations:** 1 Clinical Department of Cranio-Maxillo-Facial Surgery, Military Institute of Medicine—National Research Institute, 04-141 Warsaw, Poland; mszalwinski@wim.mil.pl (M.S.); jrozko@wim.mil.pl (J.R.); achloupek@wim.mil.pl (A.C.); 2 Department of Dental and Maxillofacial Radiology, Medical University of Warsaw, 02-097 Warsaw, Poland; 3 Ortognatyka Dr Jagielak—Private Surgical Practice, 05-090 Raszyn, Poland; mjagielak@ortognatyka.pl; 4 Clinical Department of Otolaryngology, Military Institute of Medicine—National Research Institute, 04-141 Warsaw, Poland; djurkiewicz@wim.mil.pl (D.J.); prot@wim.mil.pl (P.R.); 5 Department of Biophysics, Physiology and Pathophysiology, Medical University of Warsaw, 02-901 Warsaw, Poland; maria.sobol@wum.edu.pl

**Keywords:** virtual surgical planning, orthognathic surgery, accuracy assessment, accuracy coefficient, maxillofacial surgery, bimaxillary surgery

## Abstract

**Background:** Bimaxillary (BiMax) advancement surgeries are one of the most frequently performed procedures in the orthognathic subspecialty of craniomaxillofacial surgery. The growing digitalization of the planning process and the shift from physical to virtual settings in procedure design have allowed, among other things, for better visualization of surgeries, improved preparation, and a more profound understanding of individual anatomy. Therefore, the question of the accuracy of performed virtual planning (VP) as well as the available methods of its evaluation arises naturally. The aim of this study was to determine the accuracy of performed BiMax advancement surgeries and propose a new planning accuracy coefficient (PAC). **Methods:** A group of 35 patients who underwent BiMax surgery were included in the study. Computed tomography (CT) of the head and neck region was performed 2 weeks preoperatively and 6 months postoperatively. Acquired Digital Imaging and Communications in Medicine (DICOM) files were used to perform a VP and a 3-dimensional (3D) cephalometry analysis using IPS CASE DESIGNER^®^ software, v2.5.7.1 (KLS Martin Group, Tuttlingen, Germany). Statistical significance evaluation and basic measures of central tendency and dispersion of the analyzed variables were calculated. The accuracy of the performed planning was assessed based on the mean absolute error (MAE) between the planned and achieved cephalometric data variables. Additional assessment was performed based on the proposed PAC. **Results:** VP was found to be accurate in terms of cephalometric data assessing the height of the maxilla and mandible, the inclination of the occlusal plane, the position of the jaws in relation to the skull base, as well as overjet and overbite. There was a discrepancy in results between the classic and proposed methods of accuracy assessment in the case of several of the evaluated variables. **Conclusions:** The accuracy of the VP of BiMax advancement surgeries can be evaluated based on 3D cephalometry, and it is accurate in the assessment of the previously mentioned variables. There is a need for further analysis and potential development of the proposed PAC; however, the data obtained based on PAC are promising, and by taking into account the magnitude of planned movements, it can facilitate a fair comparison of results presented in different studies based on various assessment methods.

## 1. Introduction

Bimaxillary (BiMax) advancement surgeries are among the most common procedures performed in orthognathic surgery, a subspecialty within craniomaxillofacial clinical practice. In addition to its primary purpose of correcting disproportions of the maxillo-mandibular complex, which often result in malocclusion and facial asymmetry, it has been shown to improve upper airway volume, masticatory function, breathing, and symptoms of temporomandibular joint dysfunction, as well as enhance the patient’s psychological well-being. Patients who qualify for surgical orthognathic treatment typically undergo presurgical decompensatory treatment. After a period of orthodontic preparation, each case takes part in the surgical planning process, which is now predominantly conducted in a virtual setting [1,2,3,4,5].

The digitalization of diagnostic and planning procedures in orthognathic surgery is considered one of the most important advancements in the field over the past two decades. The technology enabled easy visualization and acknowledgement of the patient’s anatomy and possible anomalies. Reliable assessment of the upper airway, including the maxillary sinuses, morphology of the nose and soft tissues of the face, and the vascularization of the craniofacial area, contributes to a broad spectrum of diagnostic benefits. Moreover, the recent shift from fully manual protocols based on the use of articulator and face bow into partly or completely virtual planning schemes allowed for many advantages, widely presented in contemporary scientific data. Reduced planning time, improved surgical preparation and briefing, visualization of bone shifts and potential intersections, enhanced collaboration within the treatment team, the use of customized implants, and procedural repeatability are among the most frequently cited benefits. Some software also offers the ability to calculate the soft tissue movements resulting from the surgery. During the procedure, soft tissues serve mainly as an access point to the bones; their final positioning is primarily determined by the shifts in the facial bone structure. Considering the increasing emphasis on the aesthetic outcomes of the procedure, the ability to predict these results would inevitably lead to improved patient compliance and satisfaction [6,7,8,9].

A naturally arising question is the accuracy with which it is possible to plan the procedure. This information can significantly enhance the reliability of the surgery and expand knowledge about the specific outcomes resulting from the implemented surgical plan [10,11].

The purpose of this study is to analyze the accuracy of virtual surgical planning in BiMax advancement procedures using selected 3-dimensional (3D) cephalometric measurements performed on both the planned virtual surgical models and the postoperative, computed tomography (CT)-based virtual models. Additionally, we aimed to propose a better solution for measuring the accuracy of virtual planning (VP). We hypothesize that the VP performed is accurate with respect to bone tissues while simultaneously lacking the ability to predict the positioning of soft tissues.

## 2. Materials and Methods

### 2.1. Eligibility Requirements

All the patients took part in the study based on the following conditions:

The inclusion criteria: individuals aged 18 or older at the time of the surgery; patients treated with BiMax advancement surgery; date of surgery between 1 January 2022, and 31 December 2023; diagnosis of both class II and class III skeletal disorders; decompensatory treatment with fixed orthodontic braces preoperatively; completion of the virtual surgical planning; CT scans performed in our Institute’s Diagnostic Department according to the described time regimen.

The exclusion criteria: individuals under 18 years old at the time of the surgery; revision or secondary orthognathic surgery; history of previous surgical interventions of the upper airway, e.g., Functional Endoscopic Sinus Surgery (FESS), tonsillectomy, pharyngoplasty; history of craniomaxillofacial trauma; failure to adhere to the time regimen of the diagnostic procedures and follow-up appointments.

All the patients who underwent the surgery in the presented timeframe and met the presented inclusion and exclusion criteria took part in a study to address potential selection bias (Table 1).

### 2.2. Treatment

Patients underwent BiMax advancement surgery as a part of orthodontic-surgical treatment for craniofacial skeletal deformities. All the procedures, diagnostics and follow-up appointments were conducted at the Military Institute of Medicine in Warsaw, Poland. The virtual planning of the surgeries was carried out using IPS CASE DESIGNER^®^ software, v2.5.7.1 (KLS Martin Group, Tuttlingen, Germany) by P.G. and M.S. This included advancement of the maxillomandibular complex and maxillary impaction of less than 4 mm with both clockwise and counterclockwise pitch rotations. Surgical virtual models and final occlusions were based on the cone beam computed tomography (CBCT) scans (PHT-6500 scanner; Vatech, Hwaseong, Republic of Korea) of the plaster dental models, integrated into the program in a Digital Imaging and Communications in Medicine (DICOM) format. The intermediate and final surgical splints were exported as object (STL) files and printed with a medical-grade vat photopolymerization printing machine (Next Dent 5100; Next Dent, Soesterberg, The Netherlands) using surgical guide resin (Next Dent SG; Next Dent, Soesterberg, The Netherlands). Each operation was performed by the same surgical team (P.G., J.R., and M.S.) in a mandible-first, sub-spinal manner with the utilization of the classic, non-minimally invasive intraoral approach. The intraoperative intermaxillary stabilization was performed based on the Kobayashi ligatures using 4-0 metal wires and elastics. All patients received the same osteosynthesis material: system 2.0 orthognathic miniplates and screws by KLS Martin (KLS Martin Group, Tuttlingen, Germany). 

### 2.3. Data Acquisition

Patients included in the study underwent CT imaging of the head and neck area in accordance with the specified time schedule. Each scan was performed in a supine body position, using the same 64-slice CT scanner (Revolution CT 64-slice; GE Healthcare, Chicago, IL, USA) with 0.6 mm slice thickness at the Radiological Diagnostics Department of the Military Institute of Medicine. The examinations were conducted 2 weeks preoperatively and 6 months postoperatively. The patients were instructed to breathe through their nose, avoid swallowing, and maintain a stable occlusion throughout the procedure. Preoperative scans were performed with the occlusal bite wafer stabilizing the occlusion in the neutral position. Postoperative ones were performed with the occlusion stabilized in a new, constructional position with the help of the elastics. The acquired data were stored and processed in the DICOM format. 

### 2.4. Measurements

**Figure 1:**
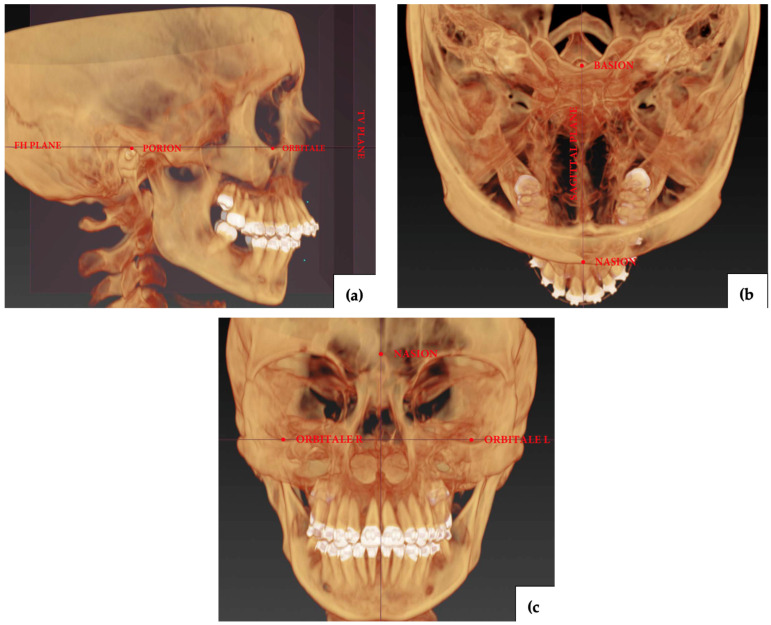
Superimposition of the 3-dimensional (3D) reconstruction model with cephalometric landmarks—pre-op (**a**) Right lateral view. (**b**) Top view. (**c**) Frontal view.

The data curation, analysis and measurements were performed in the IPS CASE DESIGNER^®^ software by P.G. and M.S. The craniofacial virtual models of each patient, both pre- and post-surgical, were superimposed in the planning software in accordance with the following reference points and lines: Frankfurt Line, Orbitale, Nasion, Basion, and Porion (Figure 1a,c). Cephalometries were performed for each CT by P.G., using the reference points listed in the Table 2 and in accordance with the build-in protocol of the planning software. All landmarks were independently double-checked by J.R. Each measurement was exported as an .CSV file into the electronic datasheet and anonymized.

### 2.5. Statistical Analysis

All calculations presented in the study were performed using Statistica 13.0 software (Dell Software Inc., Round Rock, TX, USA) and Microsoft^®^ Excel 16.89.1 software (Microsoft Corporation, Redmond, WA, USA).

For the cohort of 35 patients, the statistical power to detect significant differences between planned and 6-month postoperative outcomes at α = 0.05 was determined to be 84%.

Basic measures of central tendency and dispersion (mean ± SD, mean absolute error, median, range) were presented as descriptive statistics of the analyzed variables.

The conformity of the analyzed variables with the normal distribution was checked using the Shapiro–Wilk test. Comparisons were made between 2 assessments with the Student’s t-test for dependent variables (for variables that followed a normal distribution) or the nonparametric Wilcoxon pair test (for variables that did not follow a normal distribution), respectively. Statistical significance was set to a *p*-value < 0.05.

**Figure 2:**
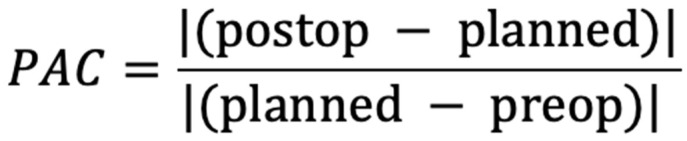
Planning Accuracy Coefficient (PAC) equation formula.

Additionally, we introduced an analysis based on a Planning Accuracy Coefficient (PAC) that relates the margin of error in procedure accuracy to the magnitude of the planned surgical shifts for individual cephalometric data sets. It is designed so that as the value of the coefficient decreases and approaches zero, the accuracy of planning increases (Figure 2).

Measures of central tendency and dispersion (mean ± SD, median, range, trimmed mean) were presented as descriptive statistics of the analyzed coefficient for each variable.

A more detailed description of PAC, together with its possible applications and limitations, is further presented in the discussion section.

### 2.6. Ethical Approval and Consent

The study was reviewed and granted an exemption of approval by the institutional ethical committee “Military Institute of Medicine–National Research Institute Bioethics Committee” (No. KB/47/24) due to its retrospective nature. The informed consent has been waived by the reviewing ethics committee due to the retrospective nature of the study and anonymization of the clinical source data. All methods were performed in accordance with relevant guidelines and regulations. The study has been conducted in accordance with the Declaration of Helsinki.

## 3. Results

A total of 35 patients took part in the study; age of the group: mean ± SD: 27.91 ± 6.63; median: 26, range: 18 to 47. Among the 35 people who took part in the study, there were 25 women and 10 men. All the included patients were Caucasian. Skeletal defect type: Class III malocclusion—23 patients and class II malocclusion—12 patients (Table 1).

The accuracy of the performed planning was assessed by mean absolute error (MAE) of the delta between the planned and achieved values of cephalometric variables, with the threshold of accuracy set at <2°/2 mm/2%. The planning was accurate for the cephalometric data describing sagittal relations of the maxilla and mandible and the base of the skull: SNA, SNB, ANB; vertical dimensions of maxilla and mandible; positioning of the teeth: overbite, overjet; and the control of the pitch movement: occlusal plane angle to FH. The planning was not accurate in the assessment of the height of the face and soft tissues of the profile: upper and lower lips, as well as the width of the face and its proportions described by the facial index.

Subsequently, each variable was assessed with the presented coefficient. The planning was considered accurate for individual parameters, with a 6% trimmed mean of the PAC value of ≤1. This threshold has been chosen as an initial starting point, corresponding to an error equal to the magnitude of virtually planned movements.

The difference in accuracy has been observed for the following selected measurements, contrary to the MAE method: facial angle, skeletal facial angle, Z-angle, lower incisor mean projection towards the TV-PL, and chin projection have been found accurate based on the PAC. Selected discussed variables, along with the values of the individual measurements, are presented in Table 3. The data for all cephalometric variables are available in Supplementary Table S1. All the landmarks used for the 3-D cephalometric measurements are presented in Table 2.

The *p*-value of <0.05 was demonstrated between the achieved and planned values in some of the cephalometric data deemed clinically accurate based on the MEA or/and PAC: ANB, SNA, facial angle, Z angle, chin projection, lower and upper incisor mean projection towards the TV-Pl, and height of the mandible.

Although a statistically significant difference was found between the mentioned planning and postoperative results for the examined parameters, it falls within the accepted clinical margin of error, and therefore the planning should still be considered accurate for those. The statistically significant difference does not quantify the actual error, which can be clinically insignificant and therefore acceptable in practice (Supplementary Table S2).

## 4. Discussion

Prior to the ongoing era of computational advancements and the introduction of 3D technology in maxillofacial surgery, conventional manual model planning for orthognathic cases was considered the gold standard in the field. Although this method has been improved and refined over time, the accuracy of the planning process has been highly dependent on the quality of manual laboratory maneuvers. The preparation of plaster models, recording and transferring the face bow, articulator setup, and splint manufacturing were highly demanding and posed as points of potential error occurrence. However, the emergence of surgical planning software and 3-D printing technologies in the last decade has enabled a gradual transformation of surgical planning methodology into a full or partial digital setting [12,13,14].

Virtual surgical planning techniques have garnered significant interest since their introduction. The emphasis has been put especially on intraoperative benefits, the assessment of the surrounding tissue changes resulting from the planned movements, and the accuracy of the performed planning itself. Another noteworthy advantage was the relative facilitation and reduction in planning time, along with the ease of case consultations between specialists from various institutions. Recent updates to available software have introduced the ability to visualize postoperative changes in the soft tissues of the face, opening the door to research on their accuracy and sparking discussions about their use during consultations, as well as the potential for further improvement of collaboration between doctors and patients [13,15,16,17].

The presented results of accuracy based on the MAE measurements are consistent with recent studies [18].

Most contemporary research evaluates accuracy by comparing the planned and obtained postoperative absolute values of specific craniofacial points in superimposition, as well as the differences between each of them. They frequently present the final results based on the calculations of the MAE for different observed variables. Another noteworthy aspect is the perceived threshold of accuracy set at <2 mm in linear differences and <4° in angular differences presented in most of the studies, regardless of the planned maneuvers and their magnitude, as well as the extent of the preexisting defect [18,19].

The use of a single, fixed threshold may oversimplify the assessment, as the difficulty of achieving surgical accuracy increases with the magnitude of the planned displacement [20,21,22]. The preset benchmark makes it easier to achieve outstanding results in groups where small operational shifts are planned. The comparison of results between different studies and populations presenting different patterns of defects is difficult and may lead to incorrect conclusions. Bengtsson, M.; et al. [23] reported a relatively high level of inaccuracy in the planning of the position of the mandible, which, according to Tondin, G.M.; et al. [19] might have been a result of qualifying patients with advanced defects, leading to more extensive planned surgical shifts.

Considering the above, in seeking to systematize the method of measuring planning accuracy, we propose the use of the PAC presented in Section 2.4. The presented coefficient takes into account the extent of the planned operation in the form of a value expressed in the denominator of the equation. As a result, with the increase in the severity of the defect and consequently the extent of the planned procedure, the margin of intraoperative error increases. It is universal and thus can be used regardless of the units of measurement of the data used to describe the preoperative, planned, and postoperative tissue positions due to their presence in both the numerator and the denominator. The use of a mathematical coefficient facilitates a fair comparison of data from different populations and surgeries performed with different methods by different surgical teams. The implementation of the presented equation into existing, widely used programs can accelerate and simplify the evaluation of results, thereby contributing to greater predictability of orthognathic surgery outcomes. It can be applied to assess existing study results and to perform a comparative analysis of the outcomes available in the literature across different populations. In addition to its use as a research tool, it may have potential applications in clinical practice. It could be implemented to perform both in- and interhospital audits. Providing a simple and quantifiable means to assess the accuracy of performed procedures can help to identify patterns of deviations and improve the consistency of outcomes and quality of performed surgeries. With further validation, the coefficient could also serve as an objective tool for accreditation and benchmarking of surgical teams. The quantitative approach could promote quality and best orthognathic practices within medical institutions.

The limitations of the presented PAC lay in the assessment of cephalometric variables related to the tissues undergoing minimal movements during the surgery as the margin of error significantly increases, resulting in high potential inaccuracies. This is especially relevant for cephalometric landmarks placed in relatively surgically stable regions with low signal-to-noise ratio. The measurement should be further tested based on future and existing studies to assess the proper margin of accuracy/inaccuracy of planning. Future validation could result in the introduction of the minimal displacement threshold to exclude the variables for which the PAC becomes unstable due to a near-zero denominator value. Stratifying variables based on the magnitude of planned movements could further improve the clinical relevance of the PAC and help mitigate the possibility of misinterpretation across different displacement ranges. The use of a 6% trimmed mean of PAC has been introduced to reduce the impact of outliers resulting from minimal surgical movement and to increase the stability of data due to the relatively small sample size.

The presented results of high accuracy regarding sagittal, vertical, and pitch planning in the maxilla and the mandible, as well as occlusion planning, based on the performed MAE measurements, are consistent with contemporary studies [18]. The higher achieved accuracy based on the PAC vs. MAE measurements regarding the variables: facial angle; skeletal facial angle; lower incisor projection towards the TV plane; Z angle; chin projection; etc. (Table 3, Supplementary Table S1) is a result of the planned, large surgical movements affecting them. The inability to make an accurate assessment of the soft tissue facial profile and proportions as well as the lip position might be caused by software imperfections in the assessment of soft tissue changes resulting from bone movements. Additionally, the differences in the postoperative response and healing, influenced by individual anatomical and biological factors, are difficult to account for in current programs. The wide variety of soft tissue profiles makes the software predictions even harder. However, future versions of existing programs may improve soft tissue predictions with the implementation of artificial intelligence (AI) and machine learning on existing datasets [24].

The authors identified a research gap in assessment methods based on 3D cephalometric data, which significantly contributed to the scientific conception of this study. Badiali, G. et al. [25] highlighted the importance of 3D cephalometry as a crucial tool for evaluating the outcomes of surgery-first orthognathic procedures. Similarly, a study by Wang, R.H. et al. [26] demonstrated that 3D cephalometry is a reliable and reproducible method in orthognathic surgery planning, emphasizing its potential benefits in outcome assessment. Cephalometric analysis is an important part of presurgical protocol [27]. Most orthognathic surgery planning software allows for cephalometric evaluation as part of the workflow. The ability to assess accuracy using an already implemented program with standard methods and available data must be considered an additional advantage of the presented system, which can be easily replicated in a different surgical and software setting. 

The choice of scanning hardware is another major consideration. This research was carried out using images obtained from medical CT hardware with the patient in the horizontal position. The advantages compared to the CBCT are higher resolution, the ability to provide a higher contrast, especially regarding soft tissues, and less image interference and distortion [28]. In contrast, the CBCT enables the patient to be scanned in a vertical position, closest to the NHP (natural head position). It is worth noting that the positioning of the patient’s head and mandible during the horizontal CT acquisition is a crucial step in achieving representative data. Based on clinical experience, the mandible-first approach seems to enable better positioning of the jaws, as the position of the condyles during the scanning does not affect the intraoperative movements [29].

The following limitations apply to this study. The limited sample size of 35 patients confines the statistical power of the calculations and has to be considered. The limited control over the collection of raw radiological data could result in inconsistent quality, potentially introducing bias and limiting the scope of the analysis. This problem has been addressed by initial data screening, excluding all studies suspected of being improperly performed.

Another limitation of the study is the lack of external validation of the proposed PAC. The accuracy threshold of 1 has been artificially set as a pragmatic starting point. As this is the first study to introduce the coefficient and present its theoretical and methodological basis, further research is needed to clarify and determine its meaningful interpretative thresholds. Such efforts should include both prospective clinical studies and analyses based on original datasets used in existing publications.

The cephalometric measurements are performed by an individual with the aid of the software, making them susceptible to human error. That introduces the potential for observer-bias and inaccuracies in the data and may impact the reliability of the results. All calculations were performed independently by two individuals in accordance with the clearly defined guidelines presented in the program in order to address this issue. However, a formal analysis such as the calculation of intra- and interobserver correlation coefficients, was not performed in this research. Future studies should include a quantitative assessment of measurement reliability to further reduce the potential bias. This limitation could also be eliminated in the future with the introduction of automated, computer-based performance of 3D cephalometry [30,31].

All cephalometric variables were presented without prioritization to maintain scientific transparency. However, certain parameters are more relevant in clinical decision-making and surgical outcome assessment. (e.g., SNA, SNB, ANB angles). Data related to parameters with less direct impact on clinical outcomes (e.g., facial index, soft tissue angles) should be interpreted with greater caution from a practical standpoint [32].

## 5. Conclusions

The accuracy of the VP of BiMax advancement surgeries can be measured using the 3D cephalometry, and it is accurate in the assessment of previously described variables, including sagittal, vertical, and pitch planning in the maxilla and the mandible, as well as occlusion planning based on the MAE measurements. 

Proposed method of assessment of the accuracy of VP using the provided planning software may contribute to the in-depth analysis of cases and the resulting improvement of surgical treatment outcomes by individual physicians.

The proposed PAC coefficient may be helpful in comparative analysis of the data provided in different studies, performed on differing populations, and may provide a fair comparison of results, regardless of the magnitude of the surgery performed. However, further studies are needed to analyze and potentially develop the coefficient, as well as to study the relationship between the size of the planned surgical movements and intraoperative error. Based on the simplicity of the proposed method for assessing accuracy and the results obtained, we believe it could become an integral part of the standard patient treatment process, enabling a more precise evaluation of treatment outcomes by surgical teams.

External validation of the formula, as well as future, larger group studies assessing the accuracy based on different methods, are needed to fully understand the clinical value and identify the potential limitations of the presented coefficient. 

## Figures and Tables

**Table 1:** Patients’ sex and type of skeletal malocclusion.

		Sex		Total
		Female	Male	
Skeletal malocclusion type	Type II	11 (31%)	1 (3%)	12 (34%)
	Type III	14 (40%)	9 (26%)	23 (66%)
Total		25 (71%)	10 (29%)	35(100%)

**Table 2:** Cephalometric landmarks.

A-point—A	Anterior Nasal spine—ANS	B-point—B	Basion—Ba	Columella constructed point—c″
Glabella—g	Gnathion—gn	Gnathion′—gn′	Gonion left—Go(l)	Gonion right—Go(r)
Incisor midpoint—I(m)	Labiale inferius—li	Labiale superius—ls	Left molar midpoint—LM(m)	Lower incisor—LI(m)
Lower incisor apex—LIapex(m)	Lower incisor apex left—LIapex(l)	Lower incisor apex right—LIapex(r)	Lower incisor left—LI(l)	Lower incisor right—LI(r)
Lower molar cusp left—LMcusp(l)	Lower molar cusp right—LMcusp(r)	Menton—Men	Nasion—N	Nasion—n
Orbitale left—Or(l)	Orbitale right—Or(r)	Pogonion—pg	Pogonion—Pog	Porion left—Po(l)
Porion midpoint—Po(m)	Porion right—Po(r)	Posterior maxillary point left—PMP(l)	Posterior maxillary point right—PMP(r)	Posterior Nasal spine—PNS
Pronasale—prn	Right molar midpoint—RM(m)	Sella—S	Stomion inferius—st(i)	Stomion superius—st(s)
Sublabiale—sl	Subnasale—sn	Subspinale—ss	Upper canine left—UC(l)	Upper canine right—UC(r)
Upper incisor—UI(m)	Upper incisor apex—UIapex(m)	Upper incisor apex left—UIapex(l)	Upper incisor apex right—UIapex(r)	Upper incisor left—UI(l)
Upper incisor right—UI(r)	Upper molar cusp left—UMcusp(l)	Upper molar cusp right—UMcusp(r)	Zygion left—zy(l)	Zygion right—zy(r)

**Table 3:** Results of statistical analysis for individual cephalometric variables. Color coding: green—accurate, red—not accurate.

	Data	ANB Angle	SNA Angle	SNB Angle	Occlusal Plane Angle to FH	Facial Angle	Skeletal Facial Angle	Height of the Face	Height of the Mandible	Height of the Maxilla
Stat.										
Delta (post-op—planned)										
Mean ± SD		−0.8 ± 1.4	−0.5 ± 1.1	0.2 ± 1.1	−0.1 ± 1.6	4.1 ± 4.1	0.9 ± 3.0	0.1 ± 2.6	0.6 ± 1.3	0.3 ± 1.2
Median (Min–Max)		0.9 (−3.8 to 1.9)	−0.6 (−2.0 to 1.5)	0.5 (−2.5 to 1.8)	−0.1 (−4.2 to 4.0)	3.7 (−2.6 to 10.7)	1.2 (−10.7 to 6.6)	0.4 (−6.0 to 7.8)	0.6 (−1.9 to 3.7)	0.3 (−2.0 to 2.8)
MAE		1.27	1.05	0.90	1.27	4.79	2.32	2.01	1.14	1.03
PAC										
Mean ± SD		0.65 ± 1.62	0.19 ± 0.13	1.29 ± 3.98	1.34 ± 2.16	1.34 ± 3.49	0.44 ± 0.42	5.25 ± 10.29	1.05 ± 1.58	1.31 ± 2.33
Median (Min–Max)		0.23 (0.02 to 8.46)	0.19 (0.01 to 0.74)	0.39 (0.02 to 24)	0.62 (0.01 to 10.05)	0.53 (0.03 to 20.08)	0.32 (0.01 to 2.1)	1.36 (0.04 to 68.75)	0.55 (0.01 to 8.06)	0.66 (0.04 to 12.0)
Trimmed Mean		0.28	0.18	0.60	0.97	0.59	0.40	3.25	0.75	1.00
	Data	Overbite	Overjet	Mentolabial angle	Nasolabial angle	Z angle	Facial index	Lower incisor mean projection towards the TV-Pl	Upper incisor mean projection towards the TV-Pl	Chin projection
Stat.										
Delta (post-op—planned)										
Mean ± SD		−0.8 ± 1.2	−0.1 ± 1.3	2.1 ± 11.0	−13.7 ± 13.1	2.3 ± 2.0	−6.5 ± 8.3	−1.6 ± 2.6	−1.7 ± 2.3	−2.2 ± 4.0
Median (Min–Max)		−0.7 (−3.6 to 2.5)	−0.1 (−2.8 to 3.0)	3.7 (−29.7 to 26.6)	−14.4 (−46.1 to 7.3)	2.7 (−3.1 to 7.6)	−5.9 (−24.5 to 13.6)	−1.5 (−8.8 to 4.3)	−1.5 (−9.0 to 3.2)	−2.7 (−11.7 to 7.7)
MAE		1.14	1.04	8.71	14.86	2.53	8.11	2.35	2.08	3.68
PAC										
Mean ± SD		0.84 ± 1.79	0.34 ± 0.73	1.55 ± 2.38	1.48 ± 1.69	1.07 ± 1.7	3.22 ± 4.17	0.79 ± 1.11	4.03 ± 7.33	0.96 ± 1.05
Median (Min–Max)		0.52 (0.05 to 10.96)	0.19 (0.01 to 4.39)	0.75 (0.01 to 11.9)	1.14 (0.09 to 10.05)	0.65 0.01 to 9.61)	2.01 (0.07 to 21.65)	0.39 0.01 to 3.04)	1.73 0.01 to 30.33)	0.62 0.05 to 5.79)
Trimmed Mean		0.53	0.21	1.09	1.22	0.74	2.58	0.59	3.31	0.80

## Data Availability

The study data are included within the manuscript and Supplementary Files. For any additional questions regarding the data, please contact the corresponding author via email.

## Supplementary Materials

The following supporting information can be downloaded at: https://www.mdpi.com/article/10.3390/jcm14103527/s1, Supplementary Table S1: Cephalometric Variables Data, Supplementary Table S2: *p*-values for cephalometric variables.
