# Supplementary material for: Evaluating Virtual Planning Accuracy in Bimaxillary Advancement Surgery: A Retrospective Study Introducing the Planning Accuracy Coefficient
Source: J Clin Med. 2025 May 18;14(10):3527. doi: 10.3390/jcm14103527 (PMC12112641; doi:10.3390/jcm14103527)
Supplement: Supplementary file 1 [file jcm-14-03527-s001.zip › Supplementary Data/Table S2.pdf]

**Results of the Wilcoxon pair test**	
**Planed vs Post-op**	p
Facial angle	<0.001
LI(m) incl. towards the TV-Pl	0.064
Nasolabial angle	<0.001
Upper Occlusal Plane Angle to TV-Pl	0.471
Chin-throat length	0.589
Interlabial gap Planned	0.896

	Results of t-pair test
**Planed vs Post-op**	p
Mentolabial angle	0.273
Skeletal facial angle	0.077
UI(m) incl, towards the TV-Pl	0.008
Upper lip angle to TV-Pl	0.005
Z angle .	<0.001
Chin projection .	0.003
Columella length .	0.015
Forehead projection towards the TV-Pl .	<0.001
Height of the chin .	0.069
Height of the face .	0.307
Height of the face (morphological) .	0.867
Height of the lower face .	<0.001
Height of the lower lip .	0.281
Height of the midface .	0.057
Height of the upper face .	0.097
Height of the upper lip .	0.005
Lower incisor mean projection towards the TV-Pl .	0.001
Lower jaw projection towards the TV-Pl .	0.001
Lower lip prominence towards the TV-Pl .	0.018
Nasal base – chin harmony .	0.001
Overbite .	0.001
Overjet .	0.788
Tip of the nose projection towards the TV-Pl .	0.001
Upper anterior facial height .	<0.001
Upper face width .	0.067
Upper incisor mean projection towards the TV-Pl .	<0.001
Upper jaw projection towards the TV-Pl .	0.020
Upper lip prominence towards the TV-Pl .	0.001
Facial index .	<0.001
ANB angle	0.002
SNA	0.008
SNB	0.370
Occlusal Plane Angle to FH	0.610
Height of the mandible	0.009
Height of the maxilla	0.201
Pog prominence towards the NB-Plane	0.401
