# Supplementary figures and images for: Evaluating Virtual Planning Accuracy in Bimaxillary Advancement Surgery: A Retrospective Study Introducing the Planning Accuracy Coefficient
Source: J Clin Med. 2025 May 18;14(10):3527. doi: 10.3390/jcm14103527 (PMC12112641; doi:10.3390/jcm14103527)

[illegible]

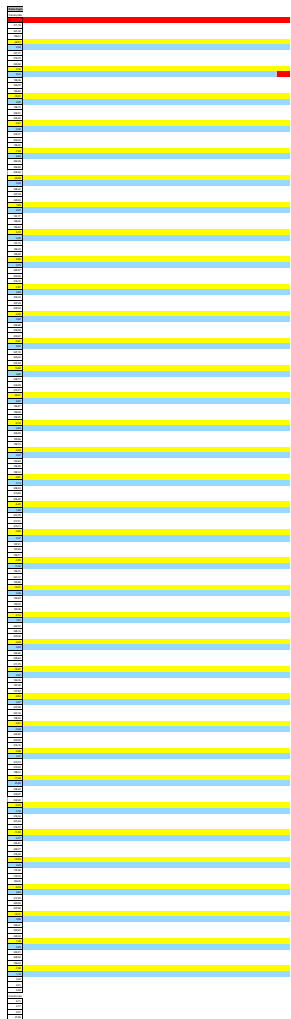

Supplement: Supplementary file 1 [file jcm-14-03527-s001.zip › Supplementary Data/Table S1.pdf]
